# First Molecular Identification of Three Clinical Isolates of Fungi Causing Mucormycosis in Honduras

**DOI:** 10.3390/idr14020031

**Published:** 2022-04-07

**Authors:** Bryan Ortiz, Isis Laínez-Arteaga, Celeste Galindo-Morales, Lilia Acevedo-Almendárez, Kateryn Aguilar, Diana Valladares, Miriam López, Gustavo Fontecha

**Affiliations:** 1 Microbiology Research Institute, Universidad Nacional Autónoma de Honduras, Tegucigalpa 11101, Honduras; bryanortiz_02@hotmail.com (B.O.); celestegalindom@gmail.com (C.G.-M.); lilia.acevedo@unah.edu.hn (L.A.-A.); kateryn.aguilar@unah.edu.hn (K.A.); 2 Bacteriology Laboratory, Hospital Mario Catarino Rivas, San Pedro Sula 21101, Honduras; izla04@gmail.com (I.L.-A.); dmgvv@hotmail.com (D.V.); milorelopez@hotmail.com (M.L.); 3 Microbiology Department, Instituto Hondureño de Seguridad Social, Tegucigalpa 11101, Honduras

**Keywords:** mucormycosis, Honduras, *Rhizopus oryzae*, *Apophysomyces ossiformis*, Mucorales, opportunistic fungal infections, coronavirus disease (COVID-19) associated mucormycosis

## Abstract

Mucormycoses are rare but serious opportunistic fungal infections caused by filamentous organisms of the order Mucorales. Here we report the first molecular identification of *Rhizopus* *oryzae* (heterotypic synonym *Rhizopus arrhizus*), *R. delemar*, and *Apophysomyces ossiformis* as the etiological agents of three cases of severe mucormycosis in Honduras. Conventional microbiological cultures were carried out, and DNA was extracted from both clinical samples and axenic cultures. The ITS ribosomal region was amplified and sequenced. Molecular tools are suitable strategies for diagnosing and identifying Mucorales in tissues and cultures, especially in middle-income countries lacking routine diagnostic strategies.

## 1. Introduction

Mucormycosis (MM) is a term that describes a set of infections produced by ubiquitous, saprophytic, and filamentous fungi of the order Mucorales [1]. MMs are angioinvasive and life-threatening opportunistic mycoses, accounting for 1.6% of invasive fungal infections predominantly among immunosuppressed patients [2]. Fungal infections caused by species of the Mucorales order can occur by inhalation of sporangiospores present in the environment or by trauma that penetrates the mucocutaneous barrier. They can also enter the body through the digestive tract after food colonization. Furthermore, environmental, or instrumental contamination may be responsible for outbreaks in the hospital [3].

MMs are generally acute, angioinvasive infections that cause diffuse, non-suppurating necrosis and severe tissue destruction. More than six forms of clinical presentation have been described according to their anatomical location: (1) rhino–orbital–cerebral, (2) pulmonary, (3) cutaneous, (4) abdominal/pelvic or gastrointestinal, (5) renal, (6) disseminated, and (7) a miscellany of other forms (endocarditis, osteomyelitis, etc.). The most frequent clinical presentations of MMs are rhino–orbital–cerebral (ROCM) and pulmonary (PM). The gastrointestinal, cutaneous, and renal clinical forms are less frequent [2,3].

The main underlying diseases that have been recognized as predisposing factors associated with MM include diabetes mellitus with or without diabetic ketoacidosis, neutropenia, hematologic and solid organ malignancies, patients receiving hematopoietic stem cells, and solid organ transplants, as well as treatment with corticosteroids [3,4]. MM is the fourth cause of systemic fungal infection, surpassed only by candidiasis, aspergillosis, and cryptococcosis. The prevalence of the causative agents of MM is approximately 10 times lower than that of the etiological agents of aspergillosis and 50–100 times lower than *Candida* spp. infections [2]. A total of 11 genera and 38 species of the order Mucorales are known to cause MM [5,6]. Species of the genera *Rhizopus*, *Rhizomucor*, *Mucor*, *Lichtheimia* (formerly *Absidia*), *Syncephalastrum,* and *Cunninghamella* are the most common causing human infections [3]. *Cokeromyces* and *Thamnostylum* are less frequently associated with MM cases worldwide [3]. 

The current pandemic by SARS-CoV-2 has caused an alarming increase in the number of MM cases, particularly in tropical regions [7,8,9]. During the pandemic alone, tens of thousands of cases of MMs have been recorded in the scientific literature in people with COVID-19, a condition that some authors have called “Coronavirus disease (COVID-19)-associated mucormycosis” (CAM) [8]. Most cases have occurred in India [10]; however, there are some cases of CAM reported in the American continent [11,12,13]. In Honduras, the first case of ROCM associated with COVID-19 was reported in July 2021. The authors did not identify with certainty the causative species, and the genus was identified morphologically as *Mucor* spp. using a conventional approach based on branched sporangiophores and the absence of rhizoid structures [14]. In December 2021, an epidemiological follow-up of 17 MM patients revealed that 11 of them were associated with COVID-19. The causative agents were not taxonomically identified in the study [15].

Due to the high mortality associated with MM, it is necessary to rely on methods that guarantee a timely diagnosis to prevent fatal outcomes. The microbiological diagnosis of MM in most developing countries faces a crucial challenge mainly due to the lack of standardized methods such as those proposed by international scientific associations in more industrialized countries [16]. The methods available for the diagnosis of MM in most clinical laboratories are traditionally based on stains and cultures, which have technical limitations [17]. For this reason, the use of molecular methods based on ribosomal ITS regions has been recognized as complementary alternatives for the diagnosis of these clinical entities [18]. One of the main advantages of techniques based on molecular biology is the precise identification at the genus and species level, which contributes to a better understanding of the epidemiology of MM.

To date, international guidelines for the treatment of MM include surgical debridement followed by treatment with liposomal Amphotericin B (AMB), along with the reversal of predisposing conditions, while Posaconazole (PCZ) is recommended as rescue therapy [19,20,21]. Moreover, Isovuconazole has been included in the treatment of invasive MM in patients who are intolerant to AMB [19]. For many years, the Mucormycetes were believed to be a homogeneous group in terms of their antifungal susceptibility profiles [22]; however, recent studies show notable differences in susceptibility between species of the order Mucorales, even within the same genus [20,21,22,23]. Therefore, the taxonomic identification of the fungi that cause MM would result in a better guide for clinicians in making therapeutic decisions, taking into account the significant differences in the profiles of sensitivity and/or resistance to antifungal agents between the species of the order Mucorales [22]. This study describes for the first time the molecular and morphological identification of the etiological agents of MM isolated from three severe clinical cases in Honduras.

## 2. Materials and Methods

We had access to three isolates resulting from serious or fatal cases of MM. One of them corresponded to a PM, the second case was a ROCM, and the third one was a cutaneous MM (CM) due to lower limb trauma. The second and third patients presented the fungal infection in a post-COVID-19 stage. The second patient had suffered from COVID-19 two months earlier and was diagnosed by RT-PCR. The third patient presented positive IgG and IgM serology at the time of diagnosis of MM. Patients with ROCM and CM had underlying diabetes.

**Figure 1:**
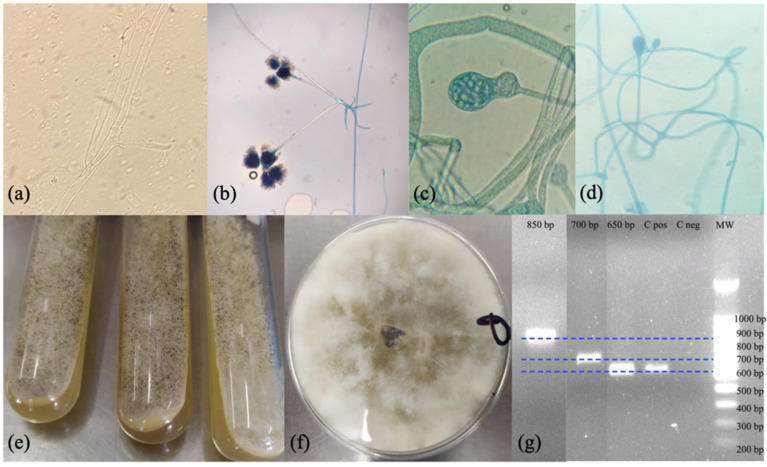
(**a**) Potassium hydroxide (KOH 10%) wet mount showing irregular, wide, and aseptate fungal hyphae. (**b**) Lactophenol cotton blue stain showing *Rhizopus* sp., 10 ×. (**c**,**d**) Lactophenol cotton blue stain showing *Apophysomyces* sp. 100 × and 40 × respectively, collected from microcultures. (**e**,**f**) Growth of white cottony colonies in PDA culture after 5 days of incubation at 28–30 °C. (**g**) Agarose gel electrophoresis of PCR products amplified using primers ITS1 and ITS4 and a 100 bp molecular weight marker (MW). The lines correspond to three different electrophoreses that have been merged into a single image.

The ROCM case met the European Organization for Research and Treatment of Cancer and the Mycoses Study Group Education and Research Consortium (EORCT/MSGER) definitions for the diagnosis of proven invasive mycoses. The cases of CM and PM were classified as probable mycosis [24,25]. The cases of PM and CM were described in patients from the city of Tegucigalpa (Social Security Honduran Institute, IHSS) while the ROCM came from the city of Bonito Oriental and was diagnosed in the Hospital Mario Catarino Rivas, San Pedro Sula. In the case of PM, a bronchoalveolar aspirate sample was collected with a sterile trap. Nasal secretions were obtained in the case of ROCM. The CM sample was obtained by debridement of the ulcerative lesion. The samples of the ROCM and PM cases were immediately sent to the laboratory for processing. Gram staining and KOH tests were performed. The samples were cultured to search for bacteria on blood agar and chocolate agar, incubated for 48 h at 37 °C. To search for fungi, potato dextrose agar (PDA) was cultured and incubated at 28–30 °C for a week under aerobic conditions. The mycological cultures were carried out in triplicate. A microculture was carried out in cases where the fungal structures did not allow their identification. In the KOH of the PM and ROCM cases, broad aseptate hyphae with right-angled branching were observed compatible with the morphology of fungi of the order Mucorales (Figure 1a). 

Due to the fungal structures observed in the KOH wet mount of the PM case, DNA extraction was carried out directly from the clinical sample using a phenol-chloroform-based approach [26]. DNA extraction from the clinical samples of the ROCM and CM cases was not performed. The ribosomal ITS region is recognized as a universal marker for the identification of fungi [27]. Likewise, sequencing of ITS regions is a suitable strategy to identify Mucorales in biological tissues as well as in culture samples [28,29,30,31]. Thus, successful amplification of this locus was obtained from the genetic material directly extracted from one clinical specimen and three mycotic cultures (Table 1). Briefly, amplifications were carried out under the following conditions. In a volume of 50 μL, 25 μL of PCR Master Mix (Promega Corp. Madison, WI, USA), 1 μL of each ITS1 and ITS4 primer (10 μM): 5′-TCC GTA GGT GAA CCT GCG G-3′/5′-TCC TCC GCT CTT ATT GAT ATG C-3′, and 1 μL of DNA (40 ng/μL). Reactions were performed with an initial denaturation step at 95 °C for 5 min, 37 cycles of 95 °C for 30 s, 55 °C for 30 s, and 72 °C for 30 s, with a final extension at 72 °C for 10 min. The amplifications were visualized on 1.5% agarose gel electrophoresis with ethidium bromide (Figure 1g). The amplified products were sequenced on both flanks with their respective primers according to Psomagen company protocols. The sequences were trimmed and edited with the Geneious^®^ 9.1.7 software.

To better understand the phylogeny of the isolates, 219 homologous sequences of Mucorales of the genera *Rhizopus*, *Mucor*, *Apophysomyces*, *Lichtheimia*, *Syncephalastrum*, *Cokeromyces*, *Thamnostylum,* and *Rhizomucor* were downloaded from GenBank. The sequences were aligned using the Muscle tool of the Geneious^®^ 9.1.7 software. The aligned sequences were edited to a length of 525 bp, and an unrooted phylogenetic tree was constructed using the Neighbor-Joining algorithm with 1000 Bootstrap replicates and no outgroup.

## 3. Results

All mycological cultures produced white cottony colonies with macroscopic morphology consistent with fungi belonging to the Mucorales order (Figure 1e,f). Lactophenol staining was performed, and the fungi isolated from the ROCM, and PM cases were morphologically identified as *Rhizopus*/*Rhizomucor* sp. by the presence of rhizoids (Figure 1b). The fungus isolated from the cutaneous ulcer in the primary culture did not produce structures that would allow identification at the genus level. Consequently, a microculture of the fungus was carried out, which allowed observing fungal structures compatible with *Apophysomyces*, *Absidia* or *Mucor* genera (Figure 1c,d).

**Figure 2:**
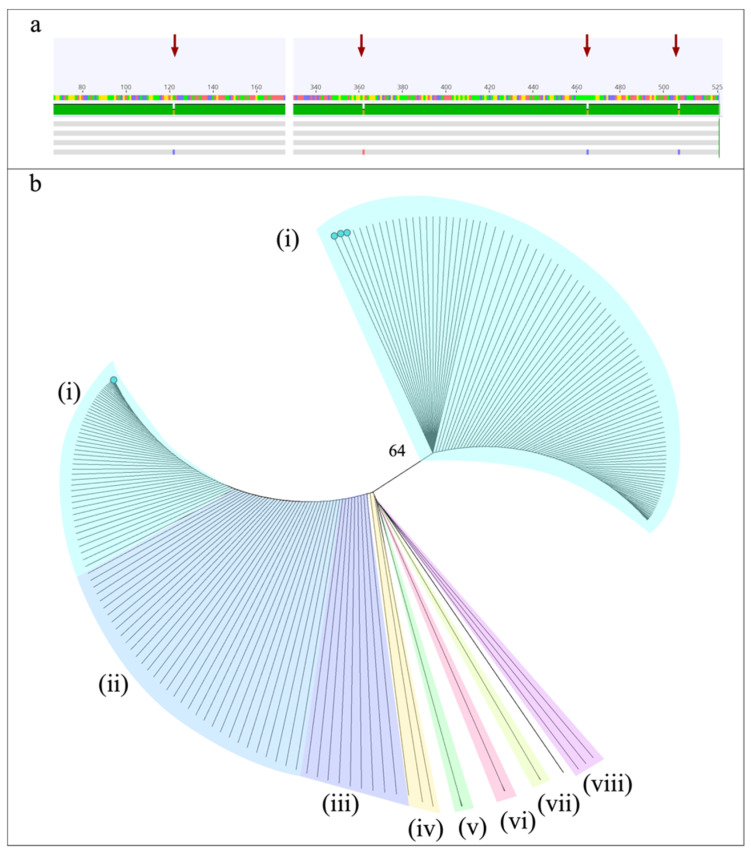
(**a**) Alignment of the four sequences obtained in this study showing four SNPs (red arrows), (**b**) Neighbor joining consensus uprooted phylogenetic tree constructed with ITS ribosomal sequences of (i) *Rhizopus arrhizus*, (ii) *R. delemar*, (iii) *R. oryzae*, (iv) *Mucor* spp., (v) *Cokeromyces* spp. and *Rhizomucor* sp., (vi) *Apophysomyces variabilis*, (vii) *Thamnostylum lucknowense*, (viii) *Lichtheimia hyalospora*. The sequences reported in this study are indicated with green dots.

Subsequently, DNA was extracted from the three fungal cultures and the ITS region was amplified and sequenced as previously described. PCR products from 650 bp to 850 bp were obtained from the cultures. The amplicons obtained from the PM clinical sample and the resulting amplicons from the three cultures were sequenced and analyzed using the NCBI BLAST tool. Four nucleotide polymorphisms were detected in a sequence of 530 nucleotides among the isolates from the PM and ROCM (Figure 2a). The microorganism causing PM was identified as *Rhizopus oryzae* (heterotypic synonym *Rhizopus arrhizus)*. However, the sequence obtained from the culture of the ROCM case was identified as *R. oryzae/R. delemar* (Table 1). The microorganism isolated from the cutaneous lesion was identified as *Apophysomyces ossiformis*. The sequences were deposited in the GenBank with the accession numbers shown in Table 1.

The cladogram obtained with the ITS ribosomal locus sequences of seven Mucorales genera shows the separation into clades of *Cokeromyces*/*Rhizomucor*, *Apophysomyces*, *Thamnostylum,* and *Lichtheimia*. The genus *Rhizopus* shows a separation of two clades with a Bootstrap of 64. One clade groups sequences from *R. arrhizus* and the second clade includes sequences from *R. arrhizus*, *R. oryzae*, *R. delemar,* and *Mucor*. The sequences obtained from the PM case are in a different clade than the sequence obtained from the ROCM case (Figure 2b). 

## 4. Discussion

MM are aggressive infections with a poor prognosis characterized by being angioinvasive and responsible for massive tissue necrosis. Due to this characteristic, these infections have been recently and incorrectly called “black fungus”, a term that must be reserved for dematiaceous fungi belonging to a different taxonomic group [32]. Mortality associated with MM can be up to 90%, particularly during disseminated infections [2,33]. The genera of fungi with the worst outcome are *Cunninghamella*, *Saksenaea*, *Rhizopus*, and *Apophysomyces* with registered mortality of 77%, 50%, 47%, and 44% respectively [33]. The main clinical presentations of MM are rhino–orbital cerebral, pulmonary, and cutaneous [3]. MM is a clinical entity considered rare in the past [2]. Recently, MM cases have increased dramatically with the current SARS-CoV-2 virus pandemic [11], mainly due to the indiscriminate use of steroids as part of the clinical management of COVID-19, in addition to concurrent risk factors such as diabetes [12]. Due to the unexpected increase in cases in the world, the Pan American Health Organization (PAHO) published an epidemiological alert of mucormycosis associated with COVID-19 (CAM), for the Americas on 11 June 2021 [34].

In this study, *Rhizopus oryzae* (heterotypic synonym *R. arrhizus*) was identified as the causative agent of two fatal cases of MM, one ROCM and the other affecting the lungs (PM). This finding is consistent with the literature where *R. oryzae* is the most common etiological agent of MM worldwide, mainly of ROCM [3,5,35]. On the other hand, *Apophysomyces ossiformis* was identified as the causative agent of a non-fatal case of CM. This finding supports the known association of *Apophysomyces* sp. with cases of CM, mainly after trauma or skin injuries [3,36,37,38]. The genus *Apophysomyces* includes six species, but only *A. elegans*, *A. mexicanus*, *A. variabilis,* and *A. ossiformis* have been reported as causing infection in humans in both immunocompetent and immunocompromised patients [39]. Infections caused by this genus are rare, with less than 3% of all MM cases worldwide [39]. Although the isolation of *Apophysomyces* is rare in the world, it has been described more frequently in India, where *A. variabilis* is the second most frequent etiological agent of MM. Furthermore, 60% of the cases of MM by *Apophysomyces* spp. in the literature are reported in India [3,5]. Cases of MM by *Apophysomyces* sp. reported in the Americas are exceptional [39]. There are very few reports in the literature describing the isolation of *A. ossiformis* from clinical samples [39], probably due to the fact that it is a recent species proposed in 2010 as part of the *A. elegans* complex [40]. 

Diabetes has been recognized as one of the main risk factors associated with MM, and more recently SARS-CoV-2 infections have been added to this list [4,10]. In this study, patients with ROCM and PM had a history of elevated blood glucose levels. Likewise, the ROCM and cutaneous cases were post-COVID-19 patients. Honduras is a country with more than 9 million inhabitants and according to PAHO and the National Diabetic Institute (INADI), about 1 million people suffer from diabetes, of which 50% are undiagnosed [41,42]. Added to this, the high morbidity of symptomatic infections by COVID-19 [43] generates a population at high risk of infection by MM-producing fungi in Honduras. In 2021, an epidemiological follow-up was published by the Ministry of Health of Honduras with the support of the PAHO and the CDC of USA, of a group of 17 patients with MM from four geographically distant hospitals in Honduras [15]. Eleven of those 17 cases were associated with COVID-19, 12 patients had underlying diabetes, and 2 had proliferative hematologic disorders. Twelve patients underwent ROCM and four presented CM. The report confirms that immunosuppressive conditions and COVID-19 infections are associated with an increase in MM cases among the population. Unfortunately, the authors identified the cases by direct microscopy, culture, or histopathology, but did not taxonomically identify the responsible fungal species.

The routine diagnosis of MM is a significant challenge for clinical laboratories. The identification and characterization of the fungal species responsible for MM are generally based on histopathology, direct examination of wet mounts, stains, and cultures [17]. Certainly, these techniques provide valuable information that contributes greatly to mycological diagnosis, but they also have limitations that should not go unnoticed. Microscopic observation of the clinical specimen with KOH and staining almost never allows identification of genus and species. On the other hand, some isolates of fungi of the Mucorales order produce *Mycelia sterilia* that do not allow their exact identification either [5]. Added to this, the cultures have a long incubation time, show low sensitivity, which in the case of MM is usually close to 50% [17,18]. Further, there are still no serological tests that contribute to the diagnosis of MM, as is the case with other fungal infections such as aspergillosis, histoplasmosis, and cryptococcosis [2,17]. Therefore, the diagnosis of MM and the definitive identification of the etiological agent traditionally based on clinic and conventional mycology must be expanded to include more recent methods based on molecular biology and mass spectrometry, increasingly accessible for laboratories in non-industrialized countries such as Honduras.

## 5. Conclusions

To our knowledge, this is the first report of *Rhizopus oryzae/delemar* and *Apophysomyces ossiformis* as etiological agents of MM in Honduras. This report sets a precedent in the use of tools based on molecular biology for the diagnosis and identification of filamentous fungi from both clinical samples and cultures in the country. The best diagnostic algorithm for MM should consider the use of molecular biology approaches in addition to conventional diagnostic techniques.

## Figures and Tables

**Table 1:** Mucormycosis-producing species identified in this study and accession numbers assigned to GenBank.

Code	Case	Origin	Species ID#	Query Cover	Per. Identity	GenBank Accession Numbers Assigned
IHSS-1	PM	Clinical sample	*Rhizopus oryzae*	100%	100%	MZ711229
IHSS-3	PM	Culture	*Rhizopus oryzae*	100%	100%	MZ711233
MCR-1	ROCM	Culture	*Rhizopus delemar/R. oryzae*	100%	100%	MZ711235
P10	CM	Culture	*Apophysomyces ossiformis*	100%	100%	OL604794

## Data Availability

Not applicable.

## References

[1] Skiada A., Lass-Floerl C., Klimko N., Ibrahim A., Roilides E., Petrikkos G. (2018). Challenges in the diagnosis and treatment of mucormycosis. Med. Mycol..

[2] Laderas J.C.P., Moreno A.P., Salido C.P., Arista J.C.R., Sicilia M.J.L. (2015). Disseminated mucormycosis in immunocompetent patients: A disease that also exists. Rev. Iberoam. Micol..

[3] Prakash H., Chakrabarti A. (2019). Global Epidemiology of Mucormycosis. J. Fungi.

[4] Prakash H., Chakrabarti A. (2021). Epidemiology of Mucormycosis in India. Microorganisms.

[5] Divakar P. (2021). Fungal Taxa Responsible for Mucormycosis/“Black Fungus” among COVID-19 Patients in India. J. Fungi.

[6] Walther G., Wagner L., Kurzai O. (2019). Updates on the Taxonomy of Mucorales with an Emphasis on Clinically Important Taxa. J. Fungi.

[7] Baskar H.C., Chandran A., Reddy C.S., Singh S. (2021). Rhino-orbital mucormycosis in a COVID-19 patient. BMJ Case Rep..

[8] Prakash H., Skiada A., Paul R., Chakrabarti A., Rudramurthy S. (2021). Connecting the Dots: Interplay of Pathogenic Mechanisms between COVID-19 Disease and Mucormycosis. J. Fungi.

[9] Revannavar S.M., Supriya P.S., Samaga L., Vineeth V. (2021). COVID-19 triggering mucormycosis in a susceptible patient: A new phenomenon in the developing world?. BMJ Case Rep..

[10] Rao V.U., Arakeri G., Madikeri G., Shah A., Oeppen R.S., Brennan P.A. (2021). COVID-19 associated mucormycosis (CAM) in India: A formidable challenge. Br. J. Oral Maxillofac. Surg..

[11] Singh A.K., Singh R., Joshi S.R., Misra A. (2021). Mucormycosis in COVID-19: A systematic review of cases reported worldwide and in India. Diabetes Metab. Syndr. Clin. Res. Rev..

[12] Guzman-Castro S., Chora-Hernandez L.D., Trujillo-Alonso G., Calvo-Villalobos I., Sanchez-Rangel A., Ferrer-Alpuin E., Ruiz-Jimenez M., Corzo-Leon D.E. (2022). COVID-19-associated mucormycosis, diabetes and steroid therapy: Experience in a single centre in Western Mexico. Mycoses.

[13] Meregildo-Rodriguez E.D., Espino-Saavedra W.G. (2021). Pediatric rhino-orbital mucormycosis. First peruvian case in times of the COVID-19 pandemic. Rev. Peru. Med. Exp. Salud Publica.

[14] Palou E.Y., Ramos M.A., Cherenfant E., Duarte A., Fuentes-Barahona I.C., Zambrano L.I., Munoz-Lara F., Montoya-Ramirez S.A., Cardona-Ortiz A.F., Valle-Reconco J.A. (2021). COVID-19 Associated Rhino-Orbital Mucormycosis Complicated by Gangrenous and Bone Necrosis-A Case Report from Honduras. Vaccines.

[15] Mejia-Santos H., Montoya S., Chacon-Fuentes R., Zielinski-Gutierrez E., Lopez B., Ning M.F., Farach N., Garcia-Coto F., Rodriguez-Araujo D.S., Rosales-Pavon K. (2021). Notes from the Field: Mucormycosis Cases During the COVID-19 Pandemic-Honduras, May-September 2021. Morb. Mortal. Wkly. Rep..

[16] Cornely O.A., Alastruey-Izquierdo A., Arenz D., Chen S.C.A., Dannaoui E., Hochhegger B., Hoenigl M., Jensen H.E., Lagrou K., Lewis R.E. (2019). Global guideline for the diagnosis and management of mucormycosis: An initiative of the European Confederation of Medical Mycology in cooperation with the Mycoses Study Group Education and Research Consortium. Lancet Infect. Dis..

[17] Skiada A., Pavleas I., Drogari-Apiranthitou M. (2020). Epidemiology and Diagnosis of Mucormycosis: An Update. J. Fungi.

[18] Schwarz P., Bretagne S., Gantier J.C., Garcia-Hermoso D., Lortholary O., Dromer F., Dannaoui E. (2006). Molecular identification of zygomycetes from culture and experimentally infected tissues. J. Clin. Microbiol..

[19] Marty F.M., Ostrosky-Zeichner L., Cornely O.A., Mullane K.M., Perfect J.R., Thompson G.R., Alangaden G.J., Brown J.M., Fredricks D.N., Heinz W.J. (2016). Isavuconazole treatment for mucormycosis: A single-arm open-label trial and case-control analysis. Lancet Infect. Dis..

[20] Macedo D., Leonardelli F., Dudiuk C., Theill L., Cabeza M.S., Gamarra S., Garcia-Effron G. (2018). Molecular Confirmation of the Linkage between the Rhizopus oryzae CYP51A Gene Coding Region and Its Intrinsic Voriconazole and Fluconazole Resistance. Antimicrob. Agents Chemother..

[21] Caramalho R., Tyndall J.D.A., Monk B.C., Larentis T., Lass-Florl C., Lackner M. (2017). Intrinsic short-tailed azole resistance in mucormycetes is due to an evolutionary conserved aminoacid substitution of the lanosterol 14alpha-demethylase. Sci. Rep..

[22] Dannaoui E. (2017). Antifungal resistance in mucorales. Int. J. Antimicrob. Agents.

[23] Macedo D., Leonardelli F., Cabeza M.S., Gamarra S., Garcia-Effron G. (2021). The natural occurring Y129F polymorphism in Rhizopus oryzae (R. arrhizus) Cyp51Ap accounts for its intrinsic voriconazole resistance. Med. Mycol..

[24] De Pauw B., Walsh T.J., Donnelly J.P., Stevens D.A., Edwards J.E., Calandra T., Pappas P.G., Maertens J., Lortholary O., Kauffman C.A. (2008). Revised Definitions of Invasive Fungal Disease from the European Organization for Research and Treatment of Cancer/Invasive Fungal Infections Cooperative Group and the National Institute of Allergy and Infectious Diseases Mycoses Study Group (EORTC/MSG) Consensus Group. Clin. Infect. Dis..

[25] Donnelly J.P., Chen S.C., Kauffman C.A., Steinbach W.J., Baddley J.W., Verweij P.E., Clancy C.J., Wingard J.R., Lockhart S.R., Groll A.H. (2020). Revision and Update of the Consensus Definitions of Invasive Fungal Disease From the European Organization for Research and Treatment of Cancer and the Mycoses Study Group Education and Research Consortium. Clin. Infect. Dis..

[26] Montes K., Ortiz B., Galindo C., Figueroa I., Braham S., Fontecha G. (2019). Identification of Candida Species from Clinical Samples in a Honduran Tertiary Hospital. Pathogens.

[27] Schoch C.L., Seifert K.A., Huhndorf S., Robert V., Spouge J.L., Levesque C.A., White M.M. (2012). Nuclear ribosomal internal transcribed spacer (ITS) region as a universal DNA barcode marker for Fungi. Proc. Natl. Acad. Sci. USA.

[28] Dannaoui E. (2009). Molecular tools for identification of Zygomycetes and the diagnosis of zygomycosis. Clin. Microbiol. Infect..

[29] Nguyen T.T.T., Jeon Y.J., Mun H.Y., Goh J., Chung N., Lee H.B. (2019). Isolation and Characterization of Four Unrecorded Mucor Species in Korea. Mycobiology.

[30] Walther G., Pawlowska J., Alastruey-Izquierdo A., Wrzosek M., Rodriguez-Tudela J.L., Dolatabadi S., Chakrabarti A., de Hoog G.S. (2013). DNA barcoding in Mucorales: An inventory of biodiversity. Persoonia.

[31] Yu J., Walther G., Van Diepeningen A.D., Gerrits Van Den Ende A.H., Li R.Y., Moussa T.A., Almaghrabi O.A., De Hoog G.S. (2015). DNA barcoding of clinically relevant Cunninghamella species. Med. Mycol..

[32] Gupta A., Sharma A., Chakrabarti A. (2021). The emergence of post-COVID-19 mucormycosis in India: Can we prevent it?. Indian J. Ophthalmol..

[33] Jeong W., Keighley C., Wolfe R., Lee W.L., Slavin M.A., Kong D.C.M., Chen S.C.-A. (2019). The epidemiology and clinical manifestations of mucormycosis: A systematic review and meta-analysis of case reports. Clin. Microbiol. Infect..

[34] Organización Panamericana de la Salud Alerta Epidemiológica Mucormicosis Asociada a la COVID-19. https://iris.paho.org/bitstream/handle/10665.2/54284/EpiUpdate11June2021_spa.pdf?sequence=2&isAllowed=y.

[35] Kermani W., Bouttay R., Belcadhi M., Zaghouani H., Ben Ali M., Abdelkefi M. (2016). ENT mucormycosis. Report of 4 cases. Eur. Ann. Otorhinolaryngol. Head Neck Dis..

[36] Al-Tarrah K., Abdelaty M., Behbahani A., Mokaddas E., Soliman H., Albader A. (2016). Cutaneous mucormycosis postcosmetic surgery. Medicine.

[37] Bonifaz A., Stchigel A.M., Guarro J., Guevara E., Pintos L., Sanchis M., Cano-Lira J.F. (2014). Primary Cutaneous Mucormycosis Produced by the New Species Apophysomyces mexicanus. J. Clin. Microbiol..

[38] Fanfair R.N., Benedict K., Bos J., Bennett S.D., Lo Y.-C., Adebanjo T., Etienne K., Deak E., Derado G., Shieh W.-J. (2012). Necrotizing Cutaneous Mucormycosis after a Tornado in Joplin, Missouri, in 2011. N. Engl. J. Med..

[39] Martinez-Herrera E., Frias-De-Leon M.G., Julian-Castrejon A., Cruz-Benitez L., Xicohtencatl-Cortes J., Hernandez-Castro R. (2020). Rhino-orbital mucormycosis due to Apophysomyces ossiformis in a patient with diabetes mellitus: A case report. BMC Infect. Dis..

[40] Alvarez E., Stchigel A.M., Cano J., Sutton D.A., Fothergill A.W., Chander J., Salas V., Rinaldi M.G., Guarro J. (2010). Molecular phylogenetic diversity of the emerging mucoralean fungus Apophysomyces: Proposal of three new species. Rev. Iberoam. Micol..

[41] Instituto Nacional del Diabético ¿Sabías que en Honduras hay 1 Millón de Diabéticos?. https://inadi.gob.hn/sitio/sabias-que-en-honduras-hay-1-millon-de-diabeticos/.

[42] OPS Honduras IV Congreso Nacional de Diabetes. https://www3.paho.org/hon/index.php?option=com_content&view=article&id=1753:iv-congreso-nacional-de-diabetes&Itemid=260.

[43] Despacho de Comunicaciones y Estrategia Presidencial Resumen de casos en Honduras: COVID19. http://covid19honduras.org.

